# Overground Walking in a Fully Immersive Virtual Reality: A Comprehensive Study on the Effects on Full-Body Walking Biomechanics

**DOI:** 10.3389/fbioe.2021.780314

**Published:** 2021-12-03

**Authors:** Brian Horsak, Mark Simonlehner, Lucas Schöffer, Bernhard Dumphart, Arian Jalaeefar, Matthias Husinsky

**Affiliations:** ^1^ Center for Digital Health and Social Innovation, St. Pölten University of Applied Sciences, St Pölten, Austria; ^2^ Department of Health, Institute of Health Sciences, St. Pölten University of Applied Sciences, St Pölten, Austria; ^3^ Department of Media and Digital Technologies, Institute of Creative∖Media/Technologies, St. Pölten University of Applied Sciences, St Pölten, Austria

**Keywords:** gait analysis, virtual environments, kinematics, kinetics, head mounted display, biomechanics, motion capturing, immersive virtual reality

## Abstract

Virtual reality (VR) is an emerging technology offering tremendous opportunities to aid gait rehabilitation. To this date, real walking with users immersed in virtual environments with head-mounted displays (HMDs) is either possible with treadmills or room-scale (overground) VR setups. Especially for the latter, there is a growing interest in applications for interactive gait training as they could allow for more self-paced and natural walking. This study investigated if walking in an overground VR environment has relevant effects on 3D gait biomechanics. A convenience sample of 21 healthy individuals underwent standard 3D gait analysis during four randomly assigned walking conditions: the real laboratory (RLab), a virtual laboratory resembling the real world (VRLab), a small version of the VRlab (VRLab−), and a version which is twice as long as the VRlab (VRLab+). To immerse the participants in the virtual environment we used a VR-HMD, which was operated wireless and calibrated in a way that the virtual labs would match the real-world. Walking speed and a single measure of gait kinematic variability (GaitSD) served as primary outcomes next to standard spatio-temporal parameters, their coefficients of variant (CV%), kinematics, and kinetics. Briefly described, participants demonstrated a slower walking pattern (−0.09 ± 0.06 m/s) and small accompanying kinematic and kinetic changes. Participants also showed a markedly increased gait variability in lower extremity gait kinematics and spatio-temporal parameters. No differences were found between walking in VRLab+ *vs.* VRLab−. Most of the kinematic and kinetic differences were too small to be regarded as relevant, but increased kinematic variability (+57%) along with increased percent double support time (+4%), and increased step width variability (+38%) indicate gait adaptions toward a more conservative or cautious gait due to instability induced by the VR environment. We suggest considering these effects in the design of VR-based overground training devices. Our study lays the foundation for upcoming developments in the field of VR-assisted gait rehabilitation as it describes how VR in overground walking scenarios impacts our gait pattern. This information is of high relevance when one wants to develop purposeful rehabilitation tools.

## 1 Introduction

Virtual reality (VR) is an emerging technology which offers access to a variety of yet partly unexplored possibilities in the field of physical rehabilitation, both from a clinical and research perspective (Canning et al., 2020). VR can be defined as a real-time computer-generated simulation of a three-dimensional environment that replaces the natural sources of stimulation of the real world by offering artificial visual, auditory, and even haptic stimuli (Steuer, 1992). VR technologies are rapidly advancing and nowadays allow users to experience virtual environments, where one can freely move and walk, via inexpensive, off-the-shelf head-mounted-displays (HMDs).

Driving factors for using VR in rehabilitation are that it offers great potential to increase intrinsic motivation, promote a more joyful and motivational exercise experience, and it was also shown to reduce the perception of pain and discomfort (Benham et al., 2019; Chen et al., 2021). As already demonstrated by Chen et al. (2021), VR might increase adherence in exercise, which is a key factor for an effective and efficient therapy. It is, therefore, not surprising that there is a constantly growing body of literature showing that VR can be a safe (Held et al., 2018) and effective tool in supporting physical rehabilitation. There are several recently published studies, including systematic reviews and meta-analysis, which underpin the potential VR offers in gait rehabilitation. It was demonstrated that VR can support gait rehabilitation for patients with Parkinson’s Disease (Mirelman et al., 2011; Lu et al., 2021), retrain gait symmetry (Shideler et al., 2021), restore function after stroke (Ahn and Hwang, 2019; Palacios-Navarro and Hogan, 2021; Peng et al., 2021), support neuro-psychomotor rehabilitation of children with cerebral palsy (de Oliveira et al., 2016), improve balance and gait in older adults (de Vries et al., 2020; Lee, 2020; Willaert et al., 2020; Delgado and Der Ananian, 2021), and is also being used to support gait training after amputation (Darter and Wilken, 2011).

In the field of gait rehabilitation, to date, VR is primarily used on treadmills. However, due to the rapidly advancing technology of HMDs (Saldana et al., 2020) there is also a growing interest in using VR to aid gait rehabilitation during overground scenarios. This would allow people to naturally walk and navigate through a virtual environment. These scenarios potentially offer yet mostly unexplored possibilities for gait rehabilitation, such as training of activities of daily living (Ahn and Hwang, 2019), obstacle crossing (Weber et al., 2021), among other environments that might not easily or safely be replicated in clinical settings. VR also allows the studying of effects of various conditions which are usually difficult to evaluate such as visual impairments, dual tasks, or environmental effects like diffuse lighting conditions, crowded places, and different room sizes (Almajid et al., 2020; Osaba et al., 2020; Taneda et al., 2021).

In light of this rapidly advancing field and the accompanying possibilities it offers for research and therapy, there is an urgent need to understand how VR environments impact individual gait patterns during overground walking. Otherwise, it will not be possible to put VR overground scenarios as a purposeful utility into both clinical and research practice for gait rehabilitation. While there are a few studies available describing the effect of walking in a VR on the treadmill (van der Krogt et al., 2014; Chan et al., 2019; Varas-Diaz et al., 2020), there is only very limited and partly contradictory research available which specifically addressed VR overground walking. For example, existing studies only partly reported adaptions to walking speed, cadence, step width, and step length next to increased variability of the same parameters (Janeh et al., 2018; Canessa et al., 2019; Martelli et al., 2019). One study reported kinematic gait adjustments in terms of increased center of mass excursion (Varas-Diaz et al., 2020). Besides the contradictory nature of the existing literature, it is limited to simple analysis of spatio-temporal gait parameters or center of mass excursion. To date, there is no information available on the effect of VR on standard full-body 3D gait analysis such as gait kinematics and kinetics and their variability. However, such analysis is necessary if the intention is to understand potential effects of VR environments on individual gait patterns during overground walking.

Therefore, the first goal of this work was to address that limitation by evaluating if overground walking in a VR environment in the context of a standard 3D gait analysis setting has a relevant effect on spatio-temporal parameters, full-body gait kinematics and kinetics, as well as on the variability of these variables.

VR offers tremendous possibilities in the design of VR environments. These environments can resemble real environments, in the case of a gait analysis setting this is most likely the gait laboratory, but they also allow to create environments with a freely chosen ‘physical dimension’ (i.e., the size of the virtual environment in real-world units) and design. From a gait analysis perspective, the walkway length is always an important factor, as the walkway length influences self-selected walking speed (Najafi et al., 2011; Stuck et al., 2020), and thus it will affect several important biomechanical variables. However, little is known if the physical dimension of the laboratory itself influences the individual gait pattern, independent by the length of the walkway. That information could be very important, especially when one wants to compare gait analysis data from different laboratories with different physical room dimensions but most likely comparable walkway lengths. Reasons for comparing or even combining data from different laboratories can be manifold, but one emerging topic is the field of data science and in particular big data and machine learning applications (Phinyomark et al., 2018). Furthermore, this information could be important to consider during the design of virtual environments for gait rehabilitation and analysis.

Thus, the second goal of our work was to investigate if the “physical dimension” of VR environments (i.e., the size of a virtual room) influences self-selected walking speed and subsequently relevant biomechanical gait variables during overground walking in VR.

## 2 Methods

### 2.1 Participants

A convenience sample of 9 male and 12 female healthy volunteers (N = 21, age: 37.62 ± 8.55 years, weight: 70.80 ± 14.86 kg, height: 169.57 ± 6.83 cm) was recruited at our University’s campus. We included volunteers aged between 18 and 65 years and excluded participants presenting any temporary or long-term conditions affecting their ability to walk. This study was approved by the local ethics committee (GS1-EK-4/682-2020) and was performed in accordance with the relevant guidelines and regulations. All participants were informed prior to the study and gave written informed consent.

### 2.2 Study Design

All participants underwent standard 3D gait analysis in four randomly assigned walking conditions: the real laboratory (RLab), a virtual laboratory resembling the real world (VRLab, 11.9 × 5.4 m), a small version of the VRlab (VRLab−, 8.7 × 5.4 m) and a version which was twice as long as the VRlab (VRLab+, 23.5 × 5.4 m). The first two conditions are related to the first goal of evaluating if overground walking in a VR environment has a relevant effect on selected gait variables. The last two virtual environments (VRLab + *vs.* VRLab−) are related to the second research goal of investigating if the “physical dimension” of the VR environment, i.e., the size of the room, influences self-selected walking speed. The walkway length, which was approximately in the center of the real and all virtual environments, was approximately 7 m long and similar for all four conditions.

### 2.3 The Virtual Overground Environment

**FIGURE 1:**
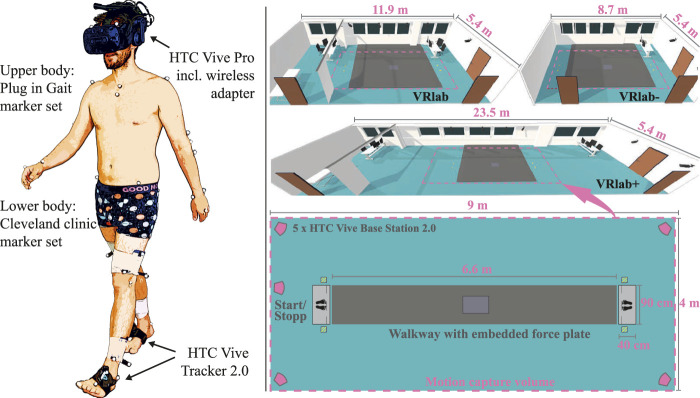
Left: The Cleveland Clinic marker set was used for the lower body and the Vicon Plug-In-Gait model for the upper body. A wireless operated HTC Vive Pro was used to deliver the VR environment. HTC Vive 2.0 trackers were used to track the positions of the feet in real-time and display them in the VR. Right: Participants walked in a random order in the real lab and three VR-versions of the lab, one resembling the real one (VRlab), one down-sized lab (VRlab−), and one over-sized lab (VRLab+). The pink dashed line shows the approximate position of the motion capture volume (of the real-world) in respect to each virtual environment.

**FIGURE 2:**
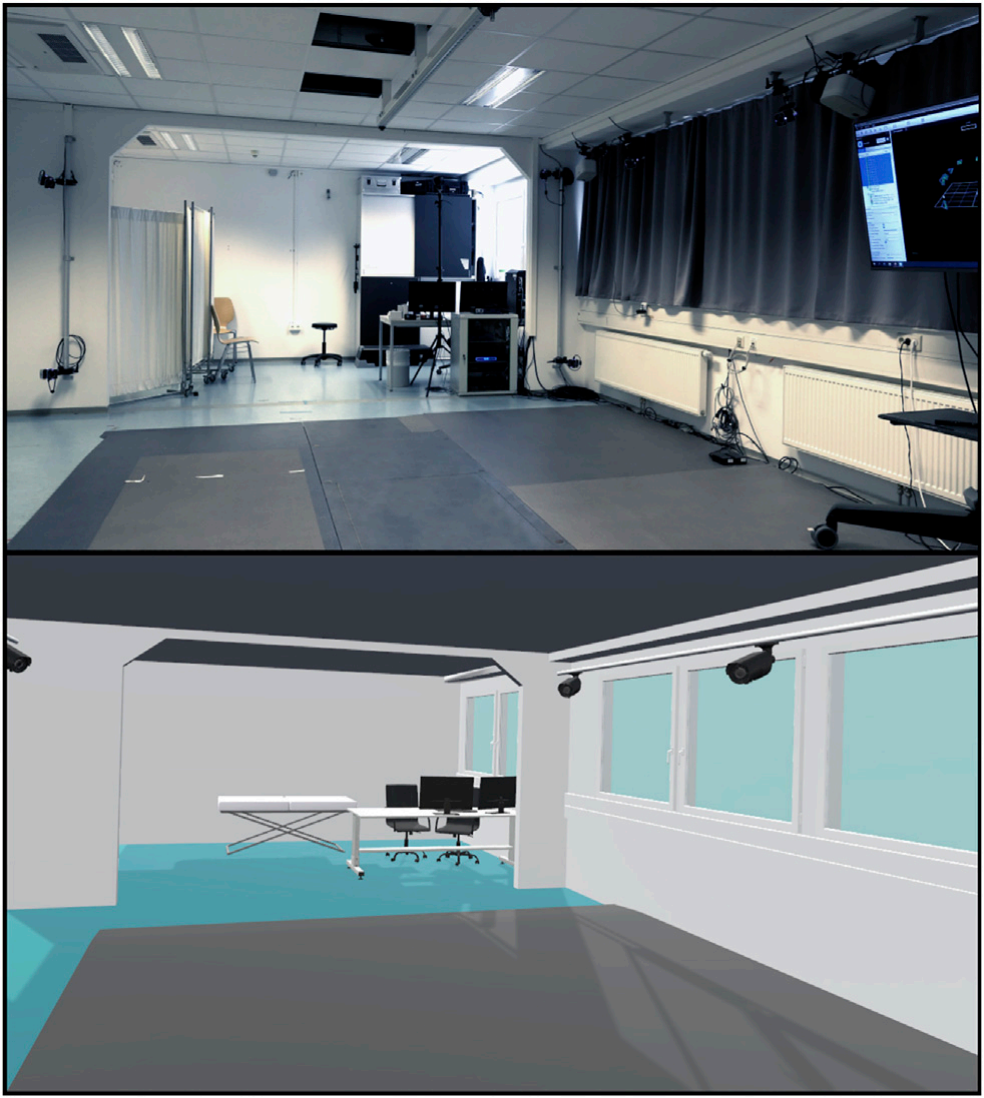
The real-world laboratory (upper picture) compared to the virtual environment resembling the real-world (lower picture) participants walked through when wearing the HMD.

The VR environment was delivered to the participants using a head-mounted-display (HMD, HTC, Vive Pro) which was operated wirelessly and calibrated to the real-world (Figures 1, 2). Participants wore the HMD on average 7 ± 1 min per VR condition and approximately 21 min in total during the study. HTC Vive 2.0 trackers were strapped to the feet to track and display the feet in VR in real-time. Five HTC Vive Lighthouses (2.0) were positioned in the laboratory to continuously track the positions of the HMD and both trackers. The real laboratory was surveyed with a laser measuring device. A virtual 3D model of the laboratory and part of its interior was created from the collected data. Based on these models, the Unity3D game engine was used for visualizing the lab in VR in real-time, as well as for the development of the application. In addition, we developed a middleware service called “Sensor Tracking Hub”. It is able to communicate with and combine data from multiple sensor input systems (in this case data received from a Vicon motion capture system and its real-time data-stream and the HTC Vive 2.0 trackers). The VR application received a continuous data stream from this middleware via the User Datagram Protocol (UDP) and applied this to a virtual 3D avatar. The setup can be easily adjusted to visualize the full movement of each participant. However, it was decided to only display virtual feet so that participants would not be too distracted during the experiment and still have a visual indication of their position in the laboratory and their movement while walking.

### 2.4 Gait Analysis and Outcome Variables

Briefly described, a 12-camera motion capture system (Nexus, 2.11, Vicon, Oxford, United Kingdom) operating at 150 Hz, and one synchronized force plate (Kistler, Winterthur, CH) sampled at 300 Hz were used to acquire standard 3D gait analysis data (3DGA). An extended Cleveland Clinic marker set (Baker, 2013) was used as a kinematic model for the lower extremity, the Vicon Plug-In-Gait model for the upper extremity (see Figure 1), and the regression equation from Davis et al. was used to determine the hip joint center (Davis et al., 1991). Ground reaction force data were filtered using a 4th order zero-lag butterworth filter with a cut-off frequency of 20 Hz. Raw kinematic trajectories were filtered using the Vicon Nexus system integrated Woltring filtering routine with a MSE value of 15. 3DGA was performed barefoot in all conditions. All data was time-normalized to 100% gait cycle (gc). Joint moments and power were normalized to body mass (N/kg, W/kg). For the conditions using the HMD, the head markers had to be replaced directly on the HMD. To account for that bias, we recorded a calibration trial for each condition where participants were asked to stand still with the head in a neutral position. The head and neck angles of the subsequent trials were then re-calibrated using that trial. In total, five clean force plate strikes per body side were recorded for each condition. For kinematic variables, however, all available steps before and after the force plate were used, resulting in 23 ± 6 steps per participant, condition, and side. One randomly chosen body side per participant was used for data analysis.

Spatio-temporal parameters, kinematic, and kinetic waveforms served as outcomes. To evaluate if walking in the VR has an effect on gait variability we also calculated a single measure of gait kinematic variability, namely GaitSD, as proposed by Sangeux et al. (2016) and calculated the coefficient of variation (CV, %) by dividing the standard deviation by the mean and multiplying it by 100 for all spatio-temporal parameters. Walking speed and GaitSD served as our primary outcomes for both research questions.

To get an impression of how participants felt during walking in the VR we asked each participant to complete a short survey immediately after the last walking session. The survey included six questions asking how they felt during walking in the VR compared to the real-world. Each question was rated on a 5-point Likert scale (see Figure 5). Participants were also asked to complete a subset of seven items of the 16-items Simulator Sickness Questionniare (SSQ) which measures symptoms such as “Headache” or “Blurred vision” on a 4-point Likert scale (none to severe) and is widely adopted for estimating motion sickness symptoms (Kennedy et al., 1993). However, the SSQ was developed for measuring experienced symptoms during a simulator and not for VR applications with HMDs. Therefore, we only used a subset of its items which fit for our purpose to measure the quality of perceived experience in VR.

### 2.5 Statistical Analysis

Basic features of the data were summarized using frequencies, means, and standard deviations, unless otherwise stated. Assumption of normality was checked by using a Kolmogorow-Smirnow Test and by inspecting the histogram of each variable. Data analysis was conducted using Python 3.8 and SPSS Statistics 26 (IBM Corporation, NY, United States).

To identify any global gait pattern changes, each 3DGA waveform was first submitted to a repeated measures ANOVA having the four conditions as within-factors from the SPM1D package (v.0.4.2) available for Python (Pataky, 2012). In case the ANOVA indicated any significant differences, additional post-hoc tests were conducted using paired-sampled t-tests in SPM. If normality of the data distribution was violated, non-parametric tests were used. To keep the amount of tests low, we only compared the RLab vs VRlab to answer research question one, and VRLab + *vs.* VRLab− to answer research question two. To reduce type I errors resulting *p*-values were Bonferroni adjusted for each pair of post hoc tests by multiplying them by 2. For the discrete spatio-temporal variables and GaitSD the same procedure was used but with the standard statistical tests for discrete values.

Based on the intrarater-intersession minimal detectable change values reported by Wilken et al. (2012) relevant differences for lower-body kinematic and kinetic variables were defined as follows: for kinematics, differences exceeding 2 degrees; for joint moments, differences exceeding 0.1 Nm/kg; for joint power, differences exceeding 0.3 W/kg. Regarding walking speed a difference of greater than 0.1 m/s was considered relevant (Bohannon and Glenney, 2014).

## 3 Results

Four out of the 21 participants reported to never have used VR-HMDs before, all others have used them only once or twice.

**FIGURE 3:**
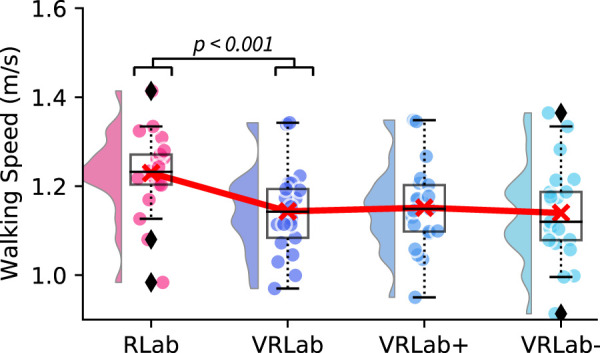
Walking speed during walking in the real lab (RLab), the virtual lab (VRLab), the over-sized (VRlab+), and down-sized (VRLab−) lab. The plot shows the data distribution (probability density function), the jittered raw data, the mean (red line), and a box plot showing quartiles where whiskers extend to the end of the data distribution except for outliers (diamonds) Allen et al. (2021).

Regarding our first primary outcome, walking speed, the repeated measures ANOVA indicated significant differences for the four walking conditions [F (3, 60) = 24.678, *p* < 0.001, eta^2^ = 0.553]. Subsequent post hoc dependent t-tests indicated a significant [t (20) = 6.18, *p* < 0.001, Cohen’s d = 1.3] decrease of 0.09 ± 0.06 m/s in walking speed when walking in the RLab compared to the VRLab, but no differences between VRLab+ and VRLab− (Figure 3). Regarding the other spatio-temproal parameters the repeated measures ANOVA and subsequent post-hoc tests indicated significant differences (*p* < 0.001) in all spatio-temporal parameters between RLab and VRLab except for step width and no differences between VRLab+ and VRLab−. See Supplementary Figure S1; Supplementary Tables S1, S2 in the supplement. Following significant results of the repeated measures ANOVA (see Supplementary Table S3 in the supplement), subsequent post-hoc tests also identified the coefficient of variation for step width (*p* < 0.001) and foot off (*p* < 0.001) as statistically increased when walking in the VRLab compared to the RLab. Step time variability slightly missed the alpha level (*p* = 0.086). No other differences were found. See Supplementary Figure S2; Supplementary Tables S3, S4 in the supplement.

**FIGURE 4:**
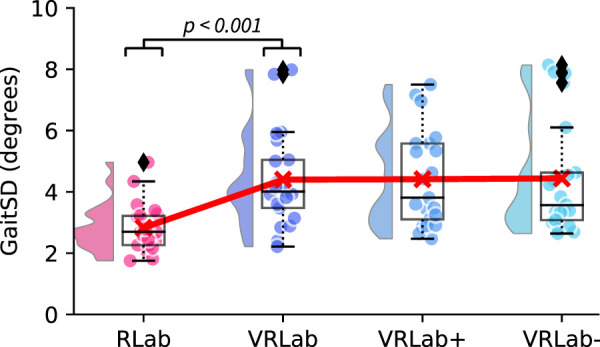
Gait variability in terms of GaitSD during walking in the real lab (RLab), the virtual lab (VRLab), the over-sized (VRlab+), and down-sized (VRLab−) lab. The plot shows the data distribution (probability density function), the jittered raw data, the mean (red line), and a box plot showing quartiles where whiskers extend to the end of the data distribution except for outliers (diamonds) Allen et al. (2021).

Regarding our second primary outcome, GaitSD, the ANOVA indicated significant differences for the four walking conditions [F (3, 60) = 11.253, *p* < 0.001, eta^2^ = 0.360]. Post hoc analysis with a Bonferroni adjustment indicated a significant [t (20) = -4.831, *p* < 0.001, Cohen’s d = 1.05] increase of on average 57% in GaitSD (Figure 4) from the RLab (2.8 ± 0.8) to the VRlab (4.4 ± 1.6), but no differences between VRlab+ (4.4 ± 1.6) and VRlab− (4.4 ± 1.9).

SPM indicated some small but significant differences in lower and upper body kinematics, joint moments, and power. However, significant differences exceeding the relevance threshold were only found for decreased plantar-flexion (−3 degrees) and sole angle during push-off (−4 degrees), as well as a decreased ankle plantar-flexion power generation during push-off (−0.4 W/kg) when walking in VR compared to the real-world. The ankle plantar flexion moment during push-off (−0.1 Nm/kg) also demonstrated a reduction slightly greater than the relevance threshold, but was not significant. Details can be found in Supplementary Figures S3–S6 in the supplement.

**FIGURE 5:**
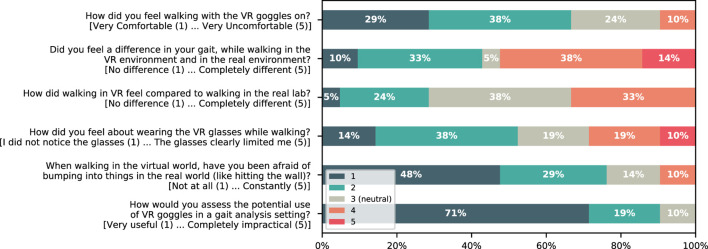
Stacked bar graphs showing the percentage of participants reporting results of the six questions about how they perceived walking in the VR compared to the real-world. The extremes of the 5-point Likert scale are described in brackets next to each question on the *y*-axis.

**FIGURE 6:**
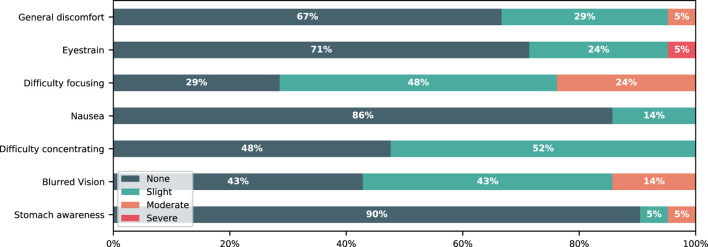
Reported results of the subset of the Simulator Sickness Questionnaire (SSQ) used to measure the perceived quality of experience during VR exposure. The stacked bar graphs show the percentage of participants reporting each question on a 4-point Likert scale.

Regarding the subset of the SSQ, most of the participants indicated that they have not noticed any or only slight symptoms. One participant (5%) reported severe eye strain along with moderate discomfort, and nausea. Five participants (24%) reported moderate difficulties in focusing and three reported moderately blurred vision (14%). For details see Figure 6. Regarding how participants perceived walking in the VR, two participants (10%) reported feeling moderately uncomfortable, 11 (52%) reported that they perceived a difference in their walking pattern while navigating through the VR, seven (33%) reported that it felt different compared to the real-world, six (29%) reported that the HMD limited them during walking, and two (10%) were moderately afraid to bump into real objects. 19 participants (90%) would like to see VR further developed in gait analysis settings (Figure 5).

## 4 Discussion

This study investigated if walking in an overground VR environment provided by an HMD has a relevant effect on selected 3D full-body gait variables in healthy individuals. We also investigated if the “physical dimension” of the VR environment influences self-selected walking speed and asked the participants to report how they perceived walking in the VR.

Participants demonstrated slight gait adaptions in spatio-temporal, kinematic, and kinetic variables during walking in the VR compared to walking in the real world. Walking speed was on average reduced by 0.09 ± 0.06 m/s (−7.3%) in the VR. Earlier research has already partly demonstrated that overground walking in VR can have an effect on spatio-temporal parameters. Martelli et al. (2019) found a decrease in stride length (∼ −4%). Janeh et al. (2017) found a decrease of walking speed (∼ −8%), step length (∼ −6%), and an increase of double support (∼ +7%) when walking in VR. Canessa et al. (2019) reported a decrease in cadence (∼ −10%). There is another particular study which analyzed whether overground VR environments that replicate freezing-of-gait (FoG) provoking situations for people with Parkinson’s disease would exacerbate gait adaptions relevant for FoG (Yamagami et al., 2020). While they have used similar motion capture techniques as in our study, they only reported a subset of spatio-temporal parameters along with step length variability. They found similar effects, such as reduced walking speed (∼ −13%), step length (∼ −11%) along with increased step width (∼ +8%) compared to the physical laboratory. However, they assessed the effect of VR overground walking in patients with impaired motor control and thus, their results cannot be directly compared to our sample of healthy individuals. In comparison to that earlier research, we have analyzed a complete set of standard spatio-temporal parameters used in clinical settings. Our results clearly support the initial findings of the preceding research with healthy participants and demonstrate that individuals change to a pattern of decreased walking speed, cadence, step/stride length, along with an increase in step/stride time, increased % double and decrease % single support (see Supplementary Tables S1, S2 for details in the supplement). In terms of statistically relevance, our results presented moderate to high effect sizes, but from a clinical perspective walking speed was slightly below our relevance threshold of 0.1 m/s (Bohannon and Glenney, 2014). Yet, in eight out of 21 participants that threshold was reached with differences ranging from 0.1 m/s to 0.23 m/s, and 0.15 ± 0.05 m/s on average.

Next to spatio-temporal adaptions, SPM indicated slight changes for lower and upper body kinematics as well as lower body joint moments and powers (see Supplementary Figures S2–S5 in the supplement). However, in almost all cases effects were small, even tough relevance thresholds were reached for plantar-flexion and sole angle during push-off, ankle plantar flexion moment during push-off, and ankle plantar-flexion power generation during push-off. These changes can be attributed to the decrease in walking speed in the VR.

Earlier research has linked HMD wear to increased neck flexion which consequently could place the musculoskeletal system of the head and neck under increased levels of stress. Knight and Baber (2007) evaluated the effects of HMDs on neck posture during HMD simulated paramedic training activities. In their study paramedics presented a greater percentage of postures with the neck flexed by greater than 20 degrees compared while immersed in VR with an HMD compared to the same condition without wearing an HMD. The HMD condition also had a greater percentage of postures that involved the neck being rotated or laterally flexed. They assumed that these changes could be either related to issues of comfort and fit, e.g., to reduce slippage, the wearers may modify their posture to better balance the HMD on the head. Their second explanation was that wearers modify their head posture to overcome a reduced field of view due to the HMD. Restricted field of view is associated with increased head movements and are assumed to have a disruptive effect on the efficient use of coordinated head and eye movements to acquire spatial information (Venturino and Wells, 1990). Our data did not indicate any changes to neck posture which is an important finding as altered neck position could limit the applicability of HMDs in clinical settings. To this end we can only make assumptions why we did not observe changes in head posture. One potential reason could be associated with the different technical advancements of the HMDs. The HMD used by Knight and Baber (2007) dates back several years whereas we have used a state-of-the-art HMD offering a much higher image resolution, a wider horizontal and vertical field of view, and a better head strap. These factors will presumably contribute to a more comfortable and natural VR experience.

Interestingly, participants also demonstrated an increased variability in spatio-temporal parameters and a markedly increase in the lower extremity gait kinematic pattern (in terms of the GaitSD) when walking in the VR. Martelli et al. (2019) already reported an increase in variability of step width (+13%) and a trend in step length variability (+24%). Also Yamagami et al. (2020) have reported an increase in step length variability (+67%) in their patients, however as mentioned above in patients with Parkinson’s disease. In line with this earlier research, we found an increase of 38% in step width variability, next to 40% in foot off variability. Our results extend that earlier results by showing that the increased variability can also be clearly seen in lower body gait kinematics (GaitSD). The demonstrated variability of spatio-temporal parameters during walking in the real world compare well to the optimal thresholds for movement performance in healthy controls described by Ravi et al. (2020). As our results show a significant increase in variability for some of the spatio-temporal parameters when walking in VR compared to the real world this could be an indication for instability induced by the VR during walking. Even tough there is also a well-documented link between reduced walking speed and increasing gait variability (Chien et al., 2015) our observed effects, especially for variability in kinematics, seem not in proportion to the expected increase due to slower walking speeds. This further corroborates the idea that the VR environment induced instability. A similar increase in variability was already reported in early research on gait adaptation for walking in VR environments on treadmills more than a decade ago (Hollman et al., 2006; Hollman et al., 2007). According to that research, individuals reduced stride length, increased step width and demonstrated pronounced variability in walking speed and step width. This was interpreted as a sign of a more conservative or cautious gait provoked by instability due to the VR environment. It was assumed that the instability may be caused by a mismatch of perception and optical flow or the fidelity of the VR system (Banton et al., 2005; Durgin et al., 2005; Sloot et al., 2014). Although we have used a much more advanced VR technology in terms of HMDs, our results point in the same direction. This seems a bit disappointing as one would assume that with better immersion technologies the walking pattern will tend to resemble a normal pattern. One reason why this was not the case might be the reduced field of view HMDs offer. Peripheral vision plays an important role in movement control and postural control and thus its limitation can influence movement (Sivak and MacKenzie, 1990; Iosa et al., 2012). Another reason might be occasionally occurring inaccuracies in alignment of the virtual to the real world caused by relative movements of the HMD to the head or blurred vision due to problems with focusing. This is also in line with the results of the questionnaires where some participants reported both, difficulty in focusing and blurred vision. These points should be addressed in future developments as they might help to increase the immersion grade while reducing unwanted effects on the gait pattern.

Regarding the results of the questionnaire, several participants reported that they perceived at least a moderate difference in their gait while walking and some also indicated that the HMD was a limiting factor during navigating through the VR. Compared to the VR-HMD, augmented reality headsets (AR-HMDs) using optical see-through displays such as Microsoft’s HoloLens 2 seem to offer advantages in that direction as AR-HMDs allow for full field of view of the real environment with digital elements superimposed to the real-world. Using AR-HMDs during overground walking could be a direction for future research. On the other hand, AR-HMDs still have the major drawback that superimposed virtual information can only be visualized with an even more narrow field of view compared to VR-HMDs. However, compared to the frequently used projection technologies for VR (such as curved screens combined with treadmills), a recent study clearly highlighted the advantages of VR-HMDs. Elor et al. (2020) compared the experience of exergaming between HMDs and a Cave Automated Virtual Environment (CAVE) and found that the HMD excelled in in-game performance, biofeedback response, and player engagement for both healthy individuals and users with cognitive disabilities. In addition, AR and VR as well HMDs are technologies that still undergo a tremendous development, so we can expect them to become even more capable, lighter, and robust in the near future.

Our second aim was to investigate if the “physical dimension” of the VR environment influences self-selected walking speed. Research has shown that walkway length influences self-selected walking speed (Najafi et al., 2011; Stuck et al., 2020), and thus it will affect several biomechanical variables. For example the recent systematic review by Stuck et al. (2020) found an overall median absolute difference of 0.04 m/s with a trend towards higher gait speed measured for longer walkway lengths compared to shorter ones. Our results did not indicate any differences in motor control adaptation between the small and the large VR environment. These results could be relevant for future studies developing virtual environments for gait rehabilitation. In addition, these results might also allow for the careful conclusion that data from different laboratories, which have similar walkway lengths but a different room-size might not be biased by the latter. This could be important especially when one wants to combine gait analysis data from different laboratories with different physical dimensions but comparable walkway lengths. Reasons for combining data from different laboratories can be manifold, but one emerging topic is the field of data science and in particular big data and machine learning applications (Phinyomark et al., 2018).

In summary, our most important findings are that people walk slightly slower and show increased gait variability when navigating in overground scenarios through virtual environments, independent by the size of the virtual room. These gait alterations need to be considered when researchers use VR environments in their investigations. In consequence, an important future direction of research should also be directed towards the identification of factors which potentially can contribute to a truly natural walking experience in VR.

There are some limitations in our work which need to be recognized. Firstly, our VR system had a limited size of the tracking area. Therefore, we had to confine the walkway length to about seven meters which is rather small and will presumably result generally in a slightly lower walking speed compared to natural walking. Secondly, our investigation is limited to healthy individuals, tough of varying age. Our study needs to be replicated with elderly people as well as with various patient groups, such as individuals with neurological disorders. Thirdly, the virtual representation of each participant in the VR was limited to their feet. Wirth et al. (2021) have recently shown that walking on a treadmill without self-representation and with different avatar self-representations, i.e., a point-cloud, a silhouette, and a humanoid representation, did not affect the gait pattern. However, the missing self-representation was rated significantly lower in terms of experienced degree of realism and the possibility to act. This could be an important factor for VR-based applications in rehabilitation as one driving factor of VR is to increase intrinsic motivation and promote a more joyful and motivational exercise experience (Chen et al., 2021). Fourthly, all walking conditions were assigned in complete random order. This means that some of our participants (N = 15/21) had one of the VR environments as their first condition. They might have developed a cautious gait already during that first condition which could have biased the following real-world walking condition to a certain extent. Lastly, since in our study the period of wearing the HMD was relatively short in comparison to the typical time spent in a rehabilitation session our findings should be cautiously used to inform the design of prolonged VR-assisted rehabilitation programs.

## 5 Conclusion

We have investigated if walking in an overground VR environment with an HMD has a relevant effect on full-body 3D gait biomechanical variables. Our results clearly demonstrate that people adopt a slightly slower walking pattern with small accompanying kinematic and kinetic changes. Most of the kinematic and kinetic differences were too small to be regarded as relevant, but reduced walking speed (−7.3%), increased lower body kinematic variability (+57%) along with increased percent double support time (+4.3%), and increased step width variability (+38%), among others, indicate gait adaptions toward a slightly more conservative or cautious gait due to instability induced by the VR environment. The “physical dimension” of the VR environment does not seem to have an impact on the gait pattern during a standard gait analysis protocol. We propose that these effects need to be taken into account in the design of VR-based overground training devices.

VR and HMDs are technologies that have been and still are undergoing a tremendous development, so we can expect them to become an even more capable tool in future to serve as a meaningful device in clinical practice and research. Our study lays the foundation for upcoming developments in the field of VR-assisted gait rehabilitation as it describes how that technology in overground walking scenarios impacts our gait patterns. This information is of high relevance when one wants to develop purposeful rehabilitation tools in future.

## Data Availability

The raw data supporting the conclusion of this article will be made available by the authors on request, without undue reservation.
