# Supplementary material for: Overground Walking in a Fully Immersive Virtual Reality: A Comprehensive Study on the Effects on Full-Body Walking Biomechanics
Source: Front Bioeng Biotechnol. 2021 Dec 3;9:780314. doi: 10.3389/fbioe.2021.780314 (PMC8693458; doi:10.3389/fbioe.2021.780314)
Supplement: Supplementary file 1 [file DataSheet1.pdf]

Supplementary Material

1 SUPPLEMENTARY PLOTS AND TABLES

The following Figures S1-S6 show the comparison of the gait analysis data for all four randomly assigned walking conditions: the real laboratory (RLab), a virtual laboratory resembling the real world (VRLab), a small version of the VRlab (VRLab-) and a version which is twice as long as the VRlab (VRLab+). The figures compare spatio-temporal parameters (Figure S1), their variability in terms of the coefficient of variation (CV) (Figure S2), lower and upper body kinematics (Figures S3 & S4), lower body joint moments (Figure S5), and powers (Figure S6) between all four conditions. In addition, Tables S1-S4 show the descriptive statistics, as well results of the repeated measures ANOVA and the subsequent post hoc analysis of the spatio-temporal parameters and their variability in terms of the coefficient of variation.

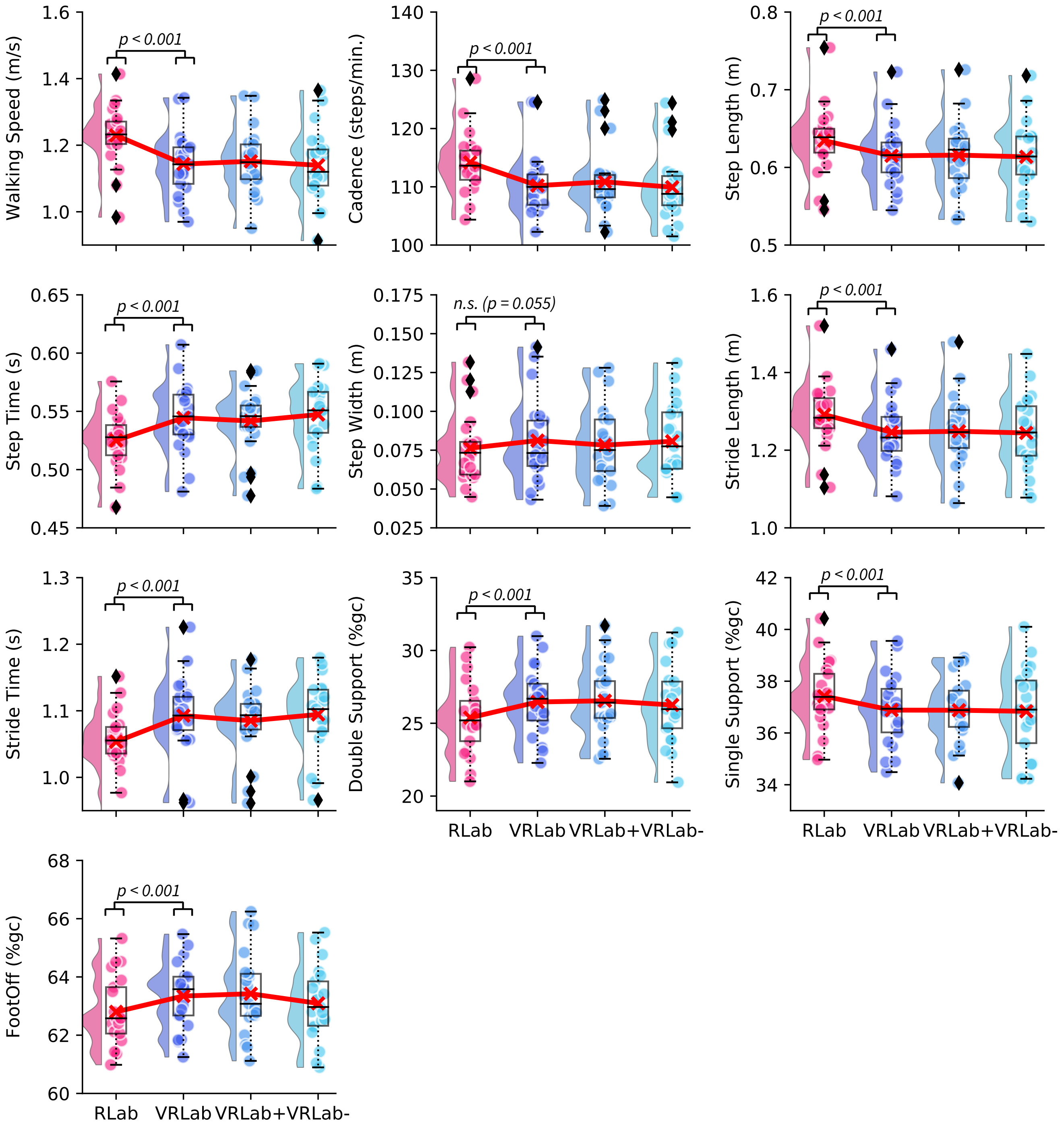

Figure S1. Spatio-temporal parameters during walking in all four randomly assigned conditions: the real laboratory (RLab), a virtual laboratory resembling the real world (VRLab), a small version of the VRlab (VRLab-) and a version which is twice as long as the VRlab (VRLab+). The plot shows the data distribution (probability density function), the jittered raw data, and a box plot showing quartiles where whiskers extend to the end of the data distribution except for outliers (diamonds) Allen et al. (2021).

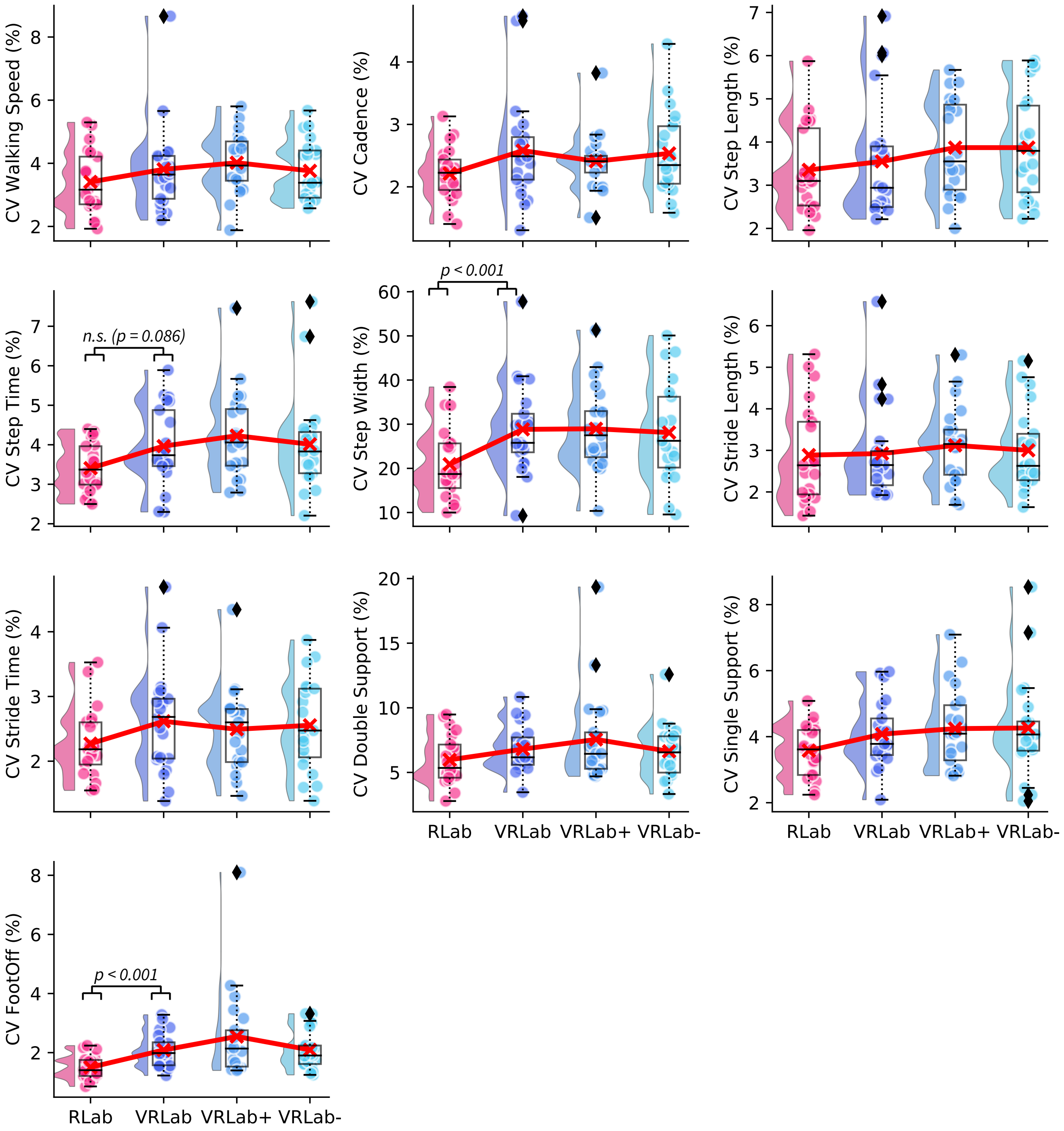

Figure S2. Variability of spatio-temporal parameters in terms of the coefficient of variation during walking in all four randomly assigned conditions: the real laboratory (RLab), a virtual laboratory resembling the real world (VRLab), a small version of the VRlab (VRLab-) and a version which is twice as long as the VRlab (VRLab+). The plot shows the data distribution (probability density function), the jittered raw data, and a box plot showing quartiles where whiskers extend to the end of the data distribution except for outliers (diamonds) Allen et al. (2021).

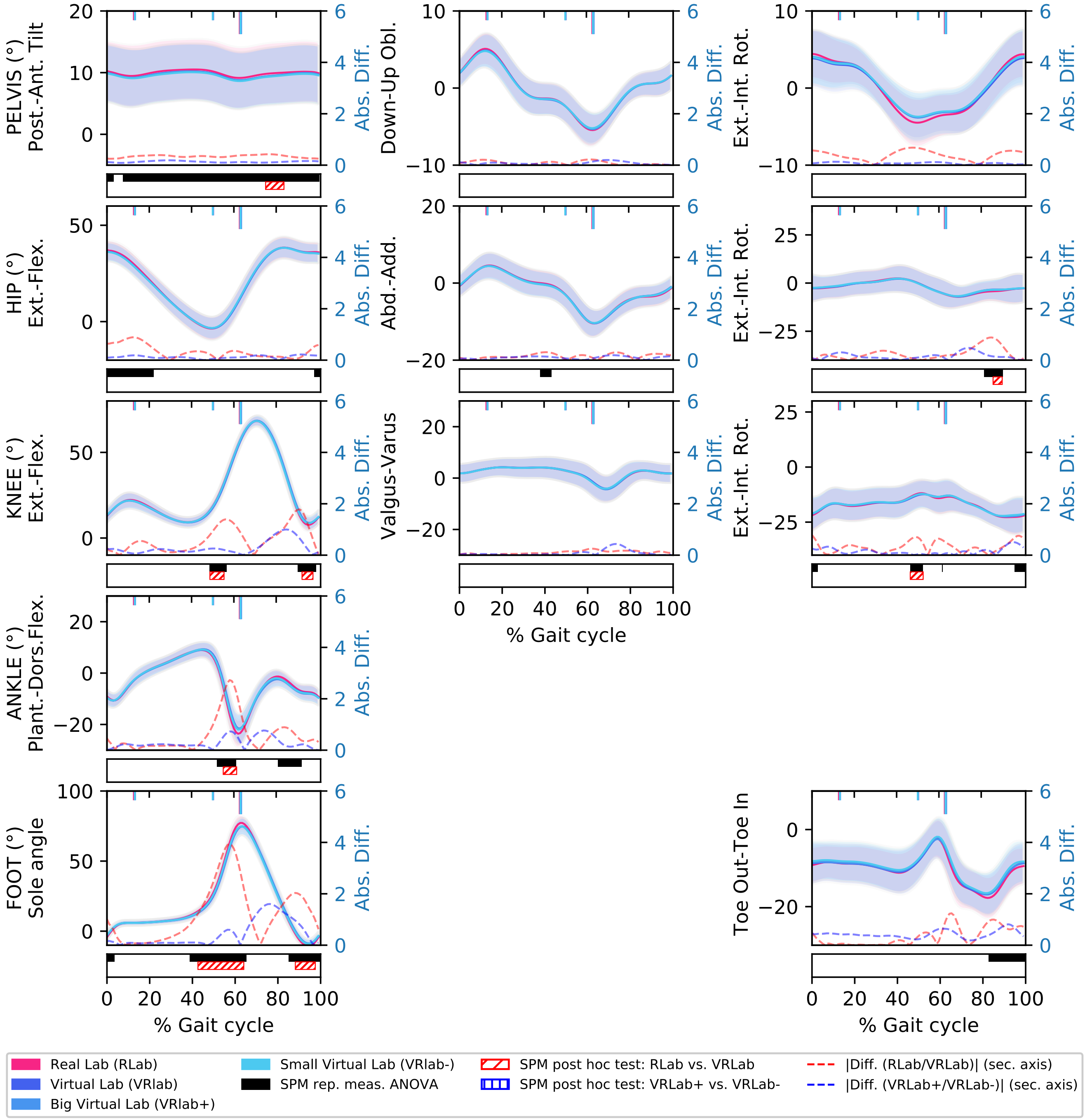

Figure S3. Lower extremity walking kinematics during all four randomly assigned conditions: the real laboratory (RLab), a virtual laboratory resembling the real world (VRLab), a small version of the VRlab (VRLab-) and a version which is twice as long as the VRlab (VRLab+). The secondary axis shows the absolute difference between the RLab vs. VRLab and VRLab+ vs. VRLab-. The bars below each subplot show significant differences indicated by the SPM repeated measures ANOVA, and subsequent pairwise post hoc tests.

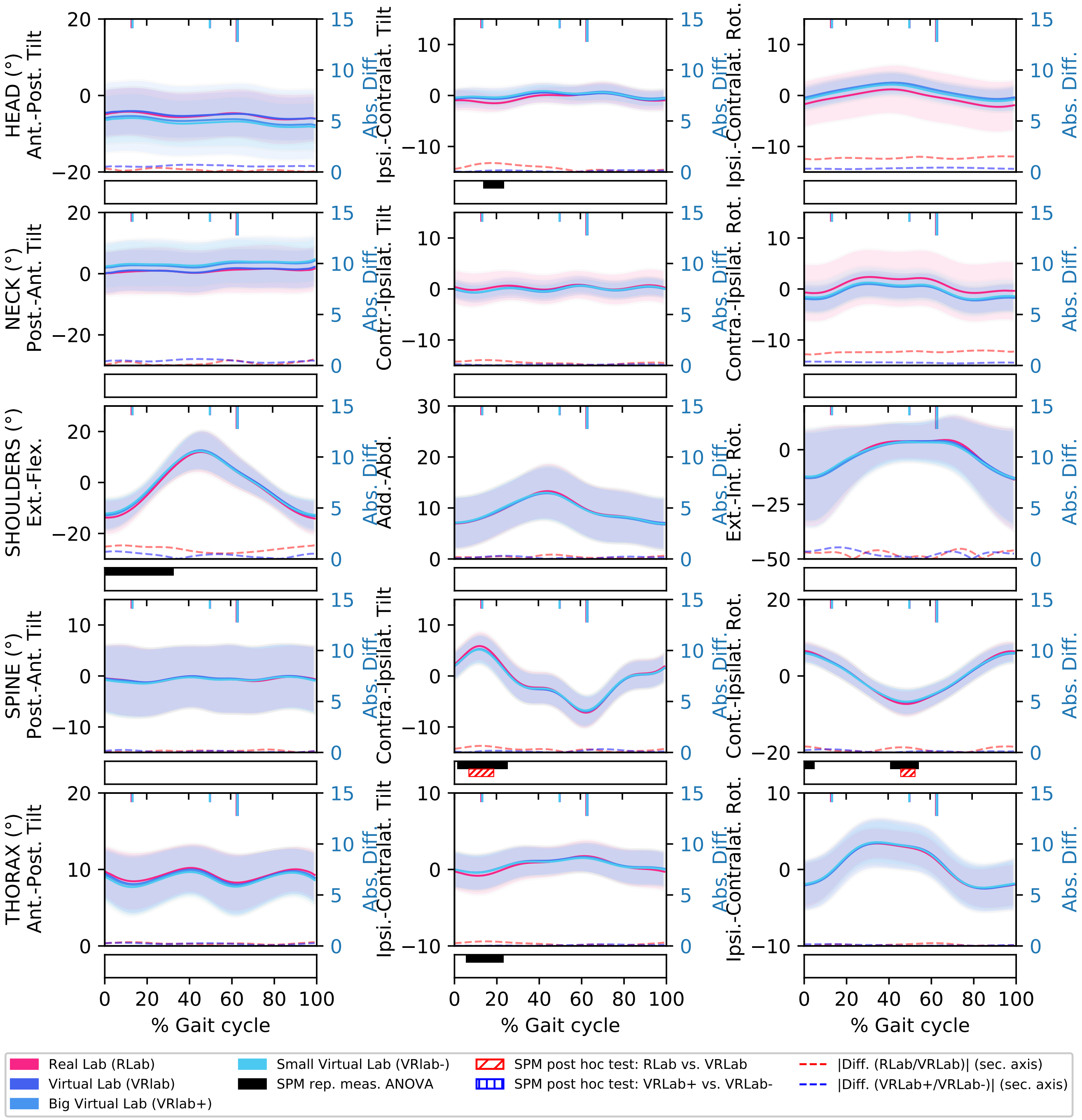

Figure S4. Upper extremity kinematics during all four randomly assigned conditions: the real laboratory (RLab), a virtual laboratory resembling the real world (VRLab), a small version of the VRlab (VRLab-) and a version which is twice as long as the VRlab (VRLab+). The secondary axis shows the absolute difference between the RLab vs. VRLab and VRLab+ vs. VRLab-. The bars below each subplot show significant differences indicated by the SPM repeated measures ANOVA, and subsequent pairwise post hoc tests.

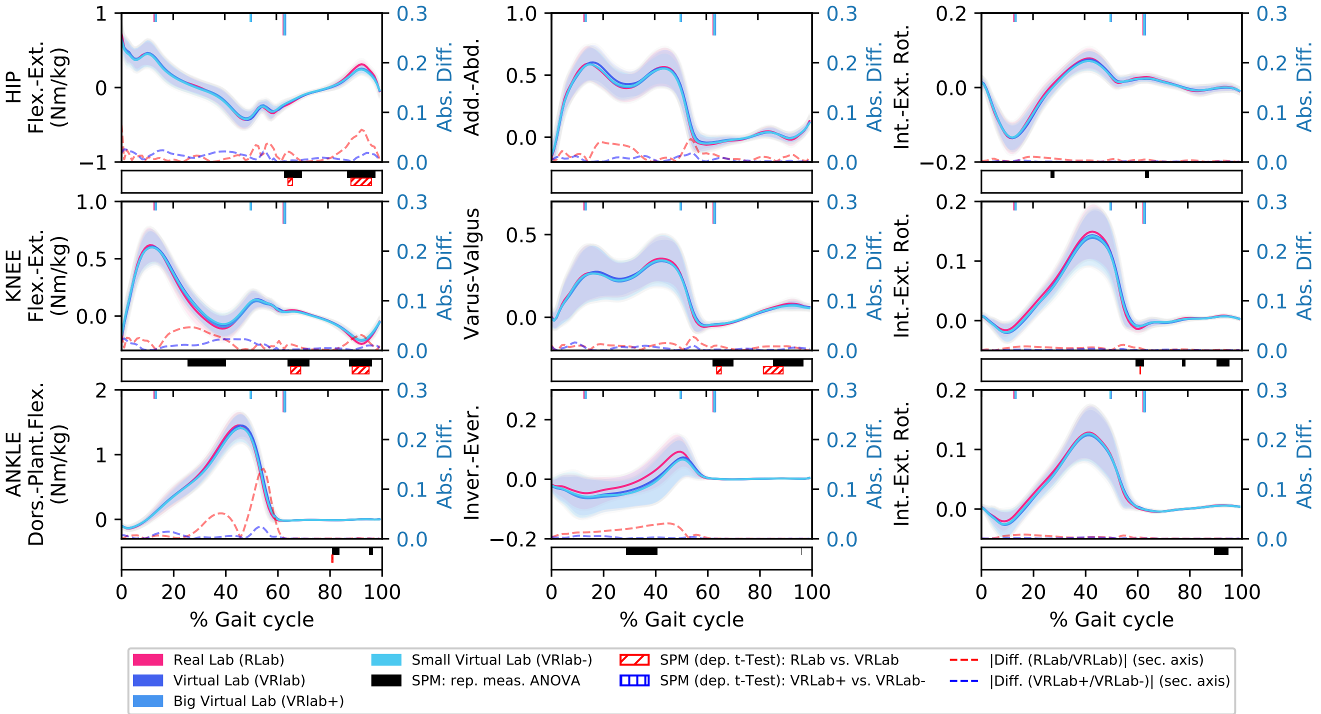

Figure S5. Lower extremity joint moments during all four randomly assigned conditions: the real laboratory (RLab), a virtual laboratory resembling the real world (VRLab), a small version of the VRlab (VRLab-) and a version which is twice as long as the VRlab (VRLab+). The secondary axis shows the absolute difference between the RLab vs. VRLab and VRLab+ vs. VRLab-. The bars below each subplot show significant differences indicated by the SPM repeated measures ANOVA, and subsequent pairwise post hoc tests.

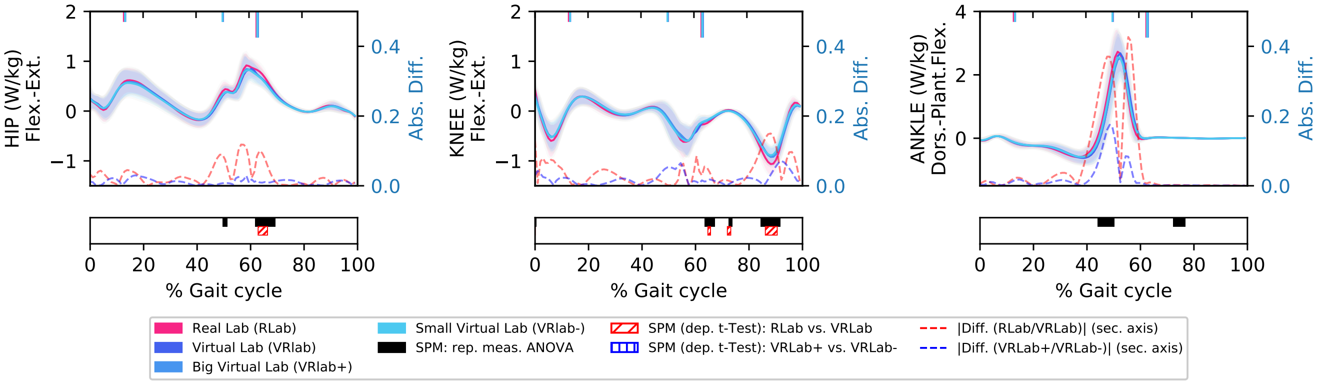

Figure S6. Lower extremity joint power during all four randomly assigned conditions: the real laboratory (RLab), a virtual laboratory resembling the real world (VRLab), a small version of the VRlab (VRLab-) and a version which is twice as long as the VRlab (VRLab+). The secondary axis shows the absolute difference between the RLab vs. VRLab and VRLab+ vs. VRLab-. The bars below each subplot show significant differences indicated by the SPM repeated measures ANOVA, and subsequent pairwise post hoc tests.

Table S1. Descriptive statistics and results of the rep. measures ANOVA for the spatio-temporal parameters between all four walking conditions: the real laboratory (RLab), a virtual laboratory resembling the real world (VRLab), a small version of the VRlab (VRLab-) and a version which is twice as long as the VRlab (VRLab+). Significant p-values are marked bold.

	**Mean (SD)**				**rep. measures Anova**			
	**RLab**	**VRLab**	**Vrlab+**	**VRLab-**	**df**	**F**	**p**	**eta^2**
WalkingSpeed (m/s)	1.23 (0.09)	1.14 (0.10)	1.15 (0.10)	1.14 (0.11)	(3, 60)	24.768	<**0.001**	0.6
Cadence (steps/min.)	114.18 (5.30)	110.21 (6.08)	110.84 (5.65)	109.93 (5.86)	(3, 60)	18.131	<**0.001**	0.5
StepLength (m)	0.63 (0.04)	0.61 (0.04)	0.62 (0.04)	0.61 (0.05)	(3, 60)	16.142	<**0.001**	0.4
StepTime (sec.)	0.53 (0.02)	0.54 (0.03)	0.54 (0.03)	0.55 (0.03)	(3, 60)	13.988	<**0.001**	0.4
StepWidth (m)	0.076 (0.023)	0.081 (0.027)	0.078 (0.025)	0.081 (0.025)	(3, 60)	2.298	0.087	0.1
StrideLength (m)	1.29 (0.09)	1.25 (0.09)	1.25 (0.09)	1.24 (0.10)	(3, 60)	20.571	<**0.001**	0.5
StrideTime (sec.)	1.05 (0.05)	1.09 (0.06)	1.09 (0.05)	1.09 (0.06)	(3, 60)	15.903	<**0.001**	0.4
DoubleSupp. (%gc)	25.38 (2.48)	26.46 (2.30)	26.54 (2.56)	26.25 (2.69)	(3, 60)	9.209	<**0.001**	0.3
SingleSupp. (%gc)	37.42 (1.34)	36.88 (1.38)	36.88 (1.29)	36.84 (1.71)	(3, 60)	6.393	<**0.001**	0.2
FootOff (%gc)	62.80 (1.20)	63.35 (1.14)	63.42 (1.41)	63.09 (1.26)	(3, 60)	5.580	**0.002**	0.2

Table S2. Mean differences and results of all spatio-temporal parameters for the paired t-test post hoc analysis for the comparison of RLab vs. VRlab and VRLab+ vs. VRLab-. Significant p-values are marked bold

	**Mean (SD) diff.**	**95%CI lower b.**	**95%CI upp. b.**	**t**	**df**	**p**	**Cohen's d**
**Rlab - VRLab**							
WalkingSpeed (m/s)	0.09 (0.06)	0.06	0.12	6.18	20	<**0.001**	1.3
Cadence (steps/min.)	3.97 (3.78)	2.25	5.69	4.81	20	<**0.001**	1.0
StepLength (m)	0.02 (0.02)	0.01	0.03	5.81	20	<**0.001**	1.3
StepTime (sec.)	-0.02 (0.02)	-0.03	-0.01	-4.60	20	<**0.001**	-1.0
StepWidth (m)	0.00 (0.01)	-0.01	0.00	-2.38	20	0.055	-0.5
StrideLength (m)	0.05 (0.03)	0.03	0.06	6.76	20	<**0.001**	1.5
StrideTime (sec.)	-0.04 (0.04)	-0.06	-0.02	-4.52	20	<**0.001**	-1.0
DoubleSupp. (%gc)	-1.08 (0.91)	-1.50	-0.66	-5.40	20	<**0.001**	-1.2
SingleSupp. (%gc)	0.54 (0.54)	0.29	0.78	4.52	20	<**0.001**	1.0
FootOff (%gc)	-0.54 (0.69)	-0.86	-0.23	-3.62	20	**0.003**	-0.8
**Rlab+ - VRLab-**							
WalkingSpeed (m/s)	0.01 (0.05)	-0.01	0.03	1.17	20	0.512	0.3
Cadence (steps/min.)	0.91 (2.30)	-0.14	1.96	1.81	20	0.169	0.4
StepLength (m)	0.00 (0.01)	0.00	0.01	0.78	20	0.895	0.2
StepTime (sec.)	-0.01 (0.02)	-0.01	0.00	-1.47	20	0.313	-0.3
StepWidth (m)	0.00 (0.01)	-0.01	0.00	-1.29	20	0.422	-0.3
StrideLength (m)	0.00 (0.03)	-0.01	0.02	0.62	20	1.000	0.1
StrideTime (sec.)	-0.01 (0.02)	-0.02	0.00	-1.73	20	0.199	-0.4
DoubleSupp. (%gc)	0.29 (1.32)	-0.31	0.89	1.00	20	1.000	0.2
SingleSupp. (%gc)	0.04 (0.86)	-0.35	0.43	0.22	20	1.000	0.0
FootOff (%gc)	0.33 (0.89)	-0.08	0.74	1.69	20	0.212	0.4

Table S3. Descriptive statistics and results of the rep. measures ANOVA for the coefficient of variation (CV) of the spatio-temporal parameters between all four walking conditions: the real laboratory (RLab). a virtual laboratory resembling the real world (VRLab). a small version of the VRlab (VRLab-) and a version which is twice as long as the VRlab (VRLab+). Significant p-values are marked bold.

	**Mean (SD)**				**rep. measures Anova**			
	**RLab**	**VRLab**	**Vrlab+**	**VRLab-**	**df**	**F**	**p**	**eta²**
CV WalkingSpeed (%)	3.4 (1.0)	3.8 (1.4)	4.0 (1.0)	3.8 (0.9)	(3, 60)	1.760	0.164	0.1
CV Cadence (%)	2.2 (0.4)	2.6 (0.8)	2.4 (0.4)	2.5 (0.7)	(3, 60)	2.598	0.060	0.1
CV StepLength (%)	3.4 (1.0)	3.6 (1.4)	3.9 (1.1)	3.9 (1.2)	(3, 60)	1.871	0.144	0.1
CV StepTime (%)	3.4 (0.6)	4.0 (1.0)	4.2 (1.1)	4.0 (1.2)	(3, 60)	3.736	**0.016**	0.2
CV StepWidth (%)	20.9 (8.1)	28.8 (10.2)	29.0 (9.5)	28.1 (11.2)	(3, 60)	12.686	**<0.001**	0.4
CV StrideLength (%)	2.9 (1.2)	2.9 (1.1)	3.1 (0.9)	3.0 (1.0)	(3, 60)	0.411	0.746	0.0
CV StrideTime (%)	2.3 (0.5)	2.6 (0.8)	2.5 (0.6)	2.6 (0.7)	(3, 60)	1.477	0.230	0.1
CV DoubleSupp. (%)	6.0 (1.9)	6.8 (1.8)	7.6 (3.5)	6.6 (2.1)	(3, 60)	1.930	0.134	0.1
CV SingleSupp. (%)	3.6 (0.8)	4.1 (1.0)	4.2 (1.2)	4.3 (1.5)	(3, 60)	2.416	0.075	0.1
CV FootOff (%)	1.5 (0.4)	2.1 (0.6)	2.5 (1.5)	2.1 (0.7)	(3, 60)	5.957	**0.001**	0.2

Table S4. Mean differences and results of the coefficient of variation (CV) for all spatio-temporal parameters for the paired t-test post hoc analysis for the comparison of RLab vs. VRlab and VRLab+ vs. VRLab-. Significant p-values are marked bold.

	**Mean (SD) diff.**	**95%CI lower b.**	**95%CI upp. b.**	**t**	**df**	**p**	**Cohen's d**
**Rlab - VRLab**							
CV WalkingSpeed (%)	-0.4 (1.3)	-1.0	0.2	-1.36	20	0.377	-0.3
CV Cadence (%)	-0.4 (0.8)	-0.8	0.0	-2.02	20	0.114	-0.4
CV StepLength (%)	-0.2 (1.3)	-0.8	0.4	-0.71	20	0.976	-0.2
CV StepTime (%)	-0.6 (1.2)	-1.1	0.0	-2.16	20	0.086	-0.5
CV StepWidth (%)	-7.9 (6.5)	-10.9	-4.9	-5.56	20	**<0.001**	-1.2
CV StrideLength (%)	0.0 (1.3)	-0.6	0.5	-0.14	20	1.000	0.0
CV StrideTime (%)	-0.3 (0.9)	-0.7	0.1	-1.79	20	0.178	-0.4
CV DoubleSupp. (%)	-0.8 (2.1)	-1.8	0.1	-1.79	20	1.000	-0.4
CV SingleSupp. (%)	-0.5 (1.1)	-1.0	0.0	-2.04	20	0.109	-0.4
CV FootOff (%)	-0.6 (0.5)	-0.8	-0.3	-5.07	20	**<0.001**	-1.1
**Rlab+ - VRLab-**							
CV WalkingSpeed (%)	0.3 (1.2)	-0.3	0.8	1.01	20	1.000	0.2
CV Cadence (%)	-0.1 (0.5)	-0.3	0.1	-1.20	20	0.485	-0.3
CV StepLength (%)	0.0 (1.0)	-0.5	0.5	0.00	20	1.000	0.0
CV StepTime (%)	0.2 (1.3)	-0.4	0.8	0.74	20	1.000	0.2
CV StepWidth (%)	0.9 (7.8)	-2.7	4.4	0.51	20	1.000	0.1
CV StrideLength (%)	0.1 (0.9)	-0.3	0.5	0.63	20	1.000	0.1
CV StrideTime (%)	-0.1 (0.6)	-0.4	0.2	-0.43	20	1.000	-0.1
CV DoubleSupp. (%)	0.9 (3.5)	-0.7	2.5	1.20	20	0.487	0.3
CV SingleSupp. (%)	0.0 (1.6)	-0.7	0.7	-0.06	20	1.000	0.0
CV FootOff (%)	0.5 (1.5)	-0.2	1.1	1.37	20	0.373	0.3

REFERENCES

Allen, M., Poggiali, D., Whitaker, K., Marshall, T. R., van Langen, J., and Kievit, R. A. (2021). Raincloud plots: A multi-platform tool for robust data visualization. *Wellcome Open Research* 4, 63. doi:10.12688/wellcomeopenres.15191.2
